# Choosing Organic Pesticides over Synthetic Pesticides May Not Effectively Mitigate Environmental Risk in Soybeans

**DOI:** 10.1371/journal.pone.0011250

**Published:** 2010-06-22

**Authors:** Christine A. Bahlai, Yingen Xue, Cara M. McCreary, Arthur W. Schaafsma, Rebecca H. Hallett

**Affiliations:** 1 School of Environmental Sciences, University of Guelph, Guelph, Ontario, Canada; 2 Department of Plant Agriculture, University of Guelph, Ridgetown, Ontario, Canada; University of Kansas, United States of America

## Abstract

**Background:**

Selection of pesticides with small ecological footprints is a key factor in developing sustainable agricultural systems. Policy guiding the selection of pesticides often emphasizes natural products and organic-certified pesticides to increase sustainability, because of the prevailing public opinion that natural products are uniformly safer, and thus more environmentally friendly, than synthetic chemicals.

**Methodology/Principal Findings:**

We report the results of a study examining the environmental impact of several new synthetic and certified organic insecticides under consideration as reduced-risk insecticides for soybean aphid (*Aphis glycines*) control, using established and novel methodologies to directly quantify pesticide impact in terms of biocontrol services. We found that in addition to reduced efficacy against aphids compared to novel synthetic insecticides, organic approved insecticides had a similar or even greater negative impact on several natural enemy species in lab studies, were more detrimental to biological control organisms in field experiments, and had higher Environmental Impact Quotients at field use rates.

**Conclusions/Significance:**

These data bring into caution the widely held assumption that organic pesticides are more environmentally benign than synthetic ones. All pesticides must be evaluated using an empirically-based risk assessment, because generalizations based on chemical origin do not hold true in all cases.

## Introduction

A public call for sustainability in agriculture has resulted in numerous government initiatives to develop environmentally friendly agricultural practices [1], [2], [3], [4], [5], [6]. In 2003, the Canadian government initiated the Pesticide Risk Reduction Program to provide infrastructure for the development and implementation of reduced-risk approaches for managing pests in crops [1]. This program, similar to ones in the UK [4] and USA [3], sought to reduce environmental risk associated with older chemical insecticides by replacing them with low risk alternatives. Though generalizations about the relative safety of natural and synthetic chemicals have been questioned in the past [7], these sustainability programs often continue to emphasize the development of organic and natural insecticides for pest control. These programs make the assumption that natural insecticides present less risk to the environment than synthetic insecticides, aligning with public opinion [8] and influential scientific papers purporting greater sustainability of organic practice [9].

The sustainability of agricultural practices is a subject of ongoing debate in the literature [10], [11], [12], [13]. Many studies have compared organic, conventional and integrated pest management (IPM) production systems as a whole, but even within a commodity system, the conclusions reached in these studies are widely divergent. A 1999 study [14] of New Zealand apple production suggested an integrated approach was more sustainable, but a 2001 study [9] of the same system in Washington favoured an organic management approach. Differing outcomes may be attributed partially to differing geography, climate and pest complexes at the two locations, but it is likely that differences in assessment methodology and the inconsistencies between specific practices classed as organic or conventional at each location were also influential in obtaining the observed results. Comparing organic, conventional and integrated agriculture is not as simple as it may initially appear [13]: each system is characterized by a suite of practices which are ideologically, rather than empirically defined [12], these systems are not mutually exclusive from each other [9], [12], and vary from region to region depending on regulations [14]. Because of these variations, generalizations about the overall sustainability of one system over another are never universal [11]. Pest management practices are often specifically highlighted in the sustainability of organic versus conventional agriculture debate, but much of the debate is fuelled by a fundamental misconception that organic farms do not use pesticides [15]. In fact, organic farms, like conventional farms, have access to a suite of pesticides [15], [16]; the primary difference is that organic regulations prohibit all synthetic (i.e.: human-made) chemicals but allow a vast array of mineral and botanical pesticides [17], whereas conventional pesticides can be both naturally and synthetically derived and are regulated individually, on a per active ingredient, per formulation basis [18].

Generalizations about the relative sustainability of one suite of practices over another are dangerous when integrated into policy: government regulations based on faulty assumptions about agricultural systems are expensive and do not effectively reduce the environmental risks they are designed to mitigate [19]. It is thus more productive, and more broadly applicable, to evaluate a given tactic for environmental sustainability on its individual properties and build policy based on results of these individual evaluations [16].

Many national and international initiatives exist to develop environmentally sustainable strategies for managing outbreaks of soybean aphid, including Agriculture and Agri-Food Canada's (AAFC) Pesticide Risk Reduction Program [1]. Soybean aphid is a severe pest of cultivated soybean in North America [20], and approximately 1.2 million hectares of soybean are cultivated each year in Canada alone [21]. Since its introduction to North America 10 years ago [20], numerous studies have examined the role of biological control agents in managing populations of aphids [22], [23], [24], [25], [26], but foliar insecticides remain necessary when populations of aphids exceed economic thresholds. The need for reduced risk pesticides in this system is profound: only two foliar insecticides are currently registered for soybean aphid control in Canada [18], one of which is currently under review for re-registration [27]. A broader suite of insecticides with varied mechanisms of action are needed to ensure effective insecticide resistance management can occur [28].

## Results

Working with AAFC, we identified four novel products to evaluate as potential reduced risk insecticides to include in integrated pest management programs for soybean aphid (Table 1). Two of these insecticides contained synthetic active ingredients, the other two are natural insecticides permitted for use in certified organic crops in Canada [17]. We included formulations of the two currently registered insecticides in the experiments as conventional controls.

**Table 1:** Insecticides evaluated for use in control of the soybean aphid.

Category	Active ingredient (ai)	Trade name (Supplier)	Mode of action	%ai	Rate per ha	EIQ*	EIQ- FUR**
Conventional (synthetic)	Cyhalothrin-λ	Matador 120E® (Syngenta)	Neurotoxin- sodium channels	13.1	83 mL	47.2	0.4
Conventional (synthetic)	Dimethoate	Lagon 480® (Cheminova)	Neurotoxin- acetylcholine esterase inhibitor	43.55	1,000 mL	33.5	12.5
Novel (synthetic)	Spirotetramat	Movento® (Bayer)	Fatty acid biosynthesis inhibitor	22.4	196 mL	34.2	1.3
Novel (synthetic)	Flonicamid	Beleaf® (FMC)	Neurotoxin- potassium channels	50	196 g	8.7	0.8
Novel (organic) ^[17]^	Mineral oil	Superior 70 oil® (UAP)	Oxygen exchange	99	11,000 mL	30.1	280.2
Novel (organic) ^[17]^	*Beauveria bassiana*	Botanigard® (Laverlam)	Entomopathogenic fungus	22	1,000 g	16.7	3.3

*per unit weight environmental impact quotient (EIQ).

**predicted EIQ-field use rating (EIQ-FUR) for a single application of the insecticide, converted to lbs/ac, as convention dictates.

We completed laboratory assays to estimate the direct contact toxicity of these insecticides to several natural enemy species when applied at field rates (Table 2). We used two of the soybean aphid's primary predator species in this study, multicoloured Asian ladybeetle *Harmonia axyridis* and insidious flower bug *Orius insidiosus* [25], [26]. There were significant differences in mortality by treatment applied for all insect groups *F_6,657_* = 325.25, *P*<.0001 for ladybeetle adults; *F_6,993_* = 1069.34, *P*<.0001 for ladybeetle larvae; *F_6,277_* = 228.11, *P*<.0001 for flower bug adults), but generally, the two currently registered insecticides were most toxic to natural enemies under laboratory conditions. The other four insecticides were much less toxic to the ladybeetle, though it was found that one of the organic insecticides, *Beauveria bassiana*, was slightly more toxic to adults, and one novel synthetic, flonicamid, was slightly more toxic to larvae than the remaining novel insecticides. The four novel pesticides all caused some mortality to the insidious flower bug, but the two organic insecticides had significantly higher toxicity than the two novel synthetic insecticides.

**Table 2:** Relative direct contact mortality of natural enemies treated with six insecticides at field rate.

	Relative H-T adjusted % mortality*		
Treatment	*Harmonia axyridis* adults	*Harmonia axyridis* larvae	*Orius insidiosus* adults
Cyhalothrin-λ	34.9b	48.2b	99.1a
Dimethoate	70.7a	99.6a	77.2b
Spirotetramat	2.0de	5.3de	20.7e
Flonicamid	0.8e	10.9c	39.5d
Mineral oil	2.6d	6.3d	60.7c
*Beauveria bassiana*	10.9c	2.7e	59.5c
Untreated control	−0.1e	−0.1f	−0.2f

*Insecticides were applied at 0.5, 1 and 2× field rate using an airbrush sprayer. Mortality was assessed at 18, 24 and 48 h post treatment for *O. insidiosus*, and every 24 h for 7d for *H. axyridis* adults and larvae. Mortality data were Henderson-Tilton adjusted ^[32]^ and subjected to a mixed model ANOVA by species and life stage, with relative rate incorporated into the model, and assessment time treated as a repeated measure. Observed mortality within a species and life stage followed by the same letter are not significantly different at α = 0.05 (LSD).

**Figure 1:**
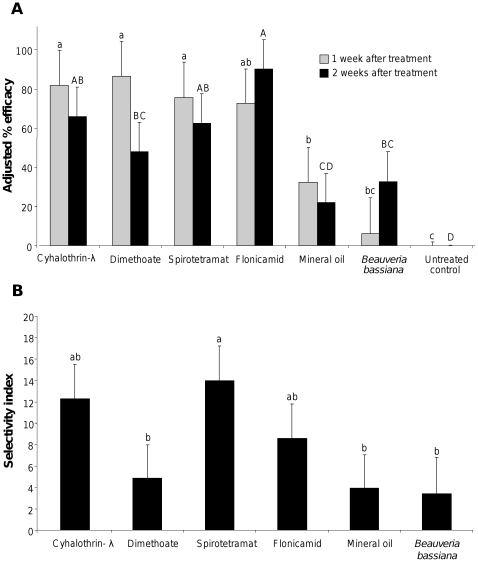
Field efficacy and selectivity observed for six insecticides for aphid control. **A) Observed efficacy.** Aphid count data were Henderson-Tilton adjusted [32] and subjected to a mixed model ANOVA by post-treatment sampling period with year of experiment, block, pass of tractor, site, and interaction terms between block and pass, block and site, and pass and site incorporated into the model. **b) Observed selectivity.** Field selectivity was determined using the natural enemy-to-aphid ratio in treatment plots, for exact calculation see Materials and Methods. Observed efficacy and selectivity within sampling period marked by the same letter are not significantly different at α = 0.05 (LSD).

**Figure 2:**
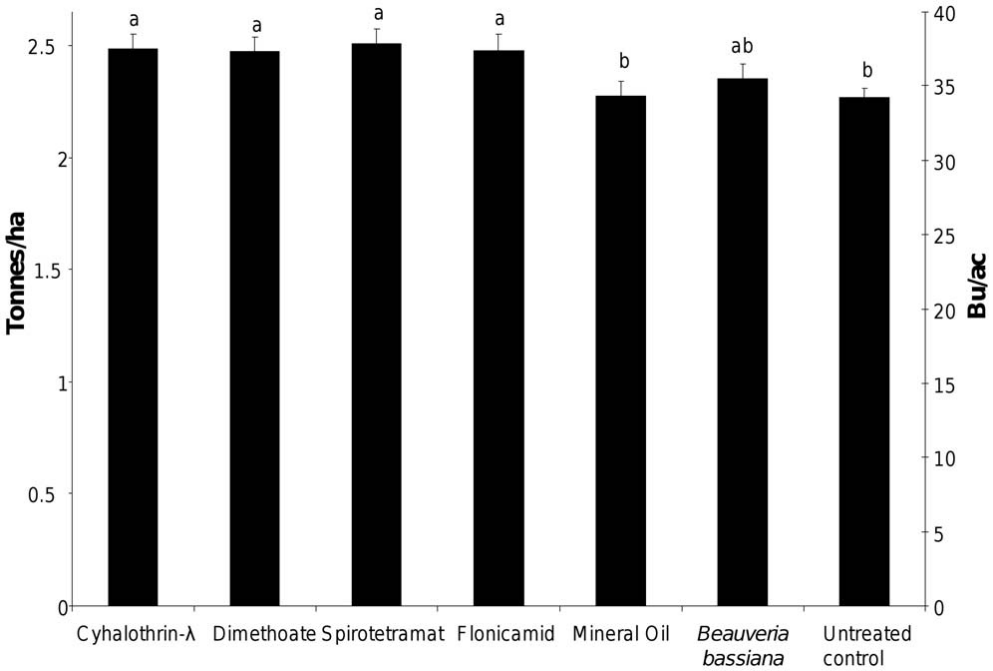
Least-square mean soybean yield in fields treated with six insecticides, 2009. Data were subjected to a mixed model ANOVA with block, site, treatment incorporated into the model. Observed yields marked by the same letter are not significantly different at α = 0.05 (LSD). Data from 2008 were excluded from analysis because of low overall aphid populations.

We conducted a two year, five site study to examine the performance of these insecticides against aphids, and selectivity with respect to natural enemies under field conditions (Fig. 1). In addition to efficacy, it is desirable for an insecticide to have a high selectivity for its target pests in order to minimize environmental impact, and to conserve biological control services provided by other organisms residing in the treated area. All synthetic insecticides had similar efficacy one week after treatment (*F_6,148_* = 7.48, *P*<0.0001), though dimethoate efficacy was reduced in the second assessment week (Fig. 1a), and yield in plots treated with synthetic insecticides did not differ significantly (*F_6,90_* = 3.51, *P* = 0.0036) (Fig. 2). The two organic insecticides had lower efficacy than the synthetic insecticides (Fig. 1a) at one week (*F_1,148_* = 25.16, *P*<0.0001) and two weeks (*F_1,121_* = 17.48, *P*<0.0001) post-treatment and did not offer significant yield protection over the untreated control (Fig. 2). Field selectivity was highest amongst synthetic insecticides, and lowest amongst organic insecticides included in this experiment (*F_1,119_* = 9.00, *P* = 0.0033), and though dimethoate had the numerically lowest selectivity amongst the synthetic insecticides, it was still numerically more selective than the organic insecticides (Fig. 1b).

Net environmental impact of applying each insecticide at given rates was estimated using an Environmental Impact Quotient analysis [29]. The per-unit-EIQ was highest for cyhalothrin-λ, a conventional synthetic insecticide (Table 1), but the EIQ-field use ratings were highest amongst the older synthetic, dimethoate, and the two organic insecticides. The high EIQ-field use rating of dimethoate was due to both a high application rate and a relatively high per-unit EIQ. The EIQ-field use rating for the mineral oil insecticide, though, was more than an order of magnitude higher than that of dimethoate, due to its relatively high per-unit-EIQ and its extremely high application rate. The remaining four insecticides had relatively low EIQ-field use ratings compared with mineral oil and dimethoate.

## Discussion

**Figure 3:**
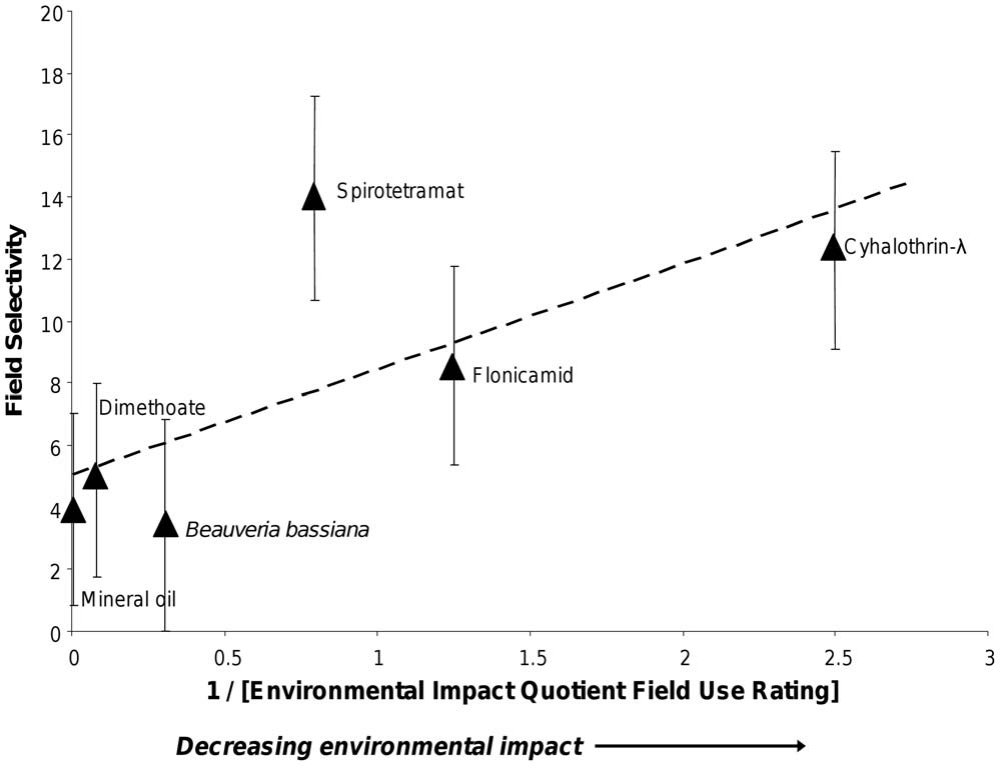
Relationship between observed field selectivity and the inverse of Environmental Impact Quotient at field rates. Field selectivities presented as least square means (± SE) of field selectivities observed at four sites in 2009. Equation of regression line is Field selectivity = (3.3±1.7)/EIQ+(0.3±3.1)+site effect, with *F_93_* = 4.23, *p* = 0.0035.

EIQ allows relative impact of various control strategies within a crop to be ranked; it is a standard method for indexing the total environmental impact of an application of a given pesticide. EIQ relies on data which is commonly available on MSDS sheets, incorporates the application rate of a pesticide, and is not site or pest-specific, so it provides a less biased estimation than other pesticide ranking systems used to quantify environmental impact [15], [30]. Because EIQ is based on a rating system and does not rely on field obtained data, some authors have criticized its use [12]. However, we found a clear inverse relationship between field selectivity and EIQ for insecticides tested in this study when applied at field rates (Fig. 3), suggesting that EIQ rankings are relevant predictors of at least some in-field parameters for environmental impact, and our results strongly support the continued use of EIQ for ranking pesticide impact. Responses of natural enemy communities are strong indicators of ecological impact of an insecticide, because they are arthropods, like the targets, and are thus likely to be biologically similar to the target of the insecticide, and because they are often found alongside the pest at the time of an insecticide application, heightening their exposure compared to other non-target organisms.

Looking at the issue empirically, our results show that with regards to environmental impact, target selectivity and efficacy, the novel synthetic insecticides we tested have better performance than organic insecticides; suggesting that certain organic management practices are not more environmentally sustainable than conventional ones. It has been purported that organic systems are not just better for the environment, but are more economically sustainable because of the price premiums associated with organic food [9]. Consumers are often willing to pay more for products they believe are produced in the most sustainable way possible, but we have shown that the organic methods available are not always the most sustainable choice. Carefully designed integrated pest management systems are likely the best strategy for minimizing environmental impact of agriculture: where certified organic systems may reject the technology with the smallest environmental impact based on ideology [11], IPM maintains the flexibility to incorporate any strategy empirically determined to have the smallest impact. In fact, it has been argued that studies which have concluded that IPM has a greater impact than organic management [9] have simply tested a poorly designed IPM strategy in which the efficacy and impact of individual tactics included in the program were not effectively examined [12], did not accurately reflect IPM practice, or employed biased methods of evaluation [15]. Though IPM practice does not typically come with price premiums associated with the production of organic food, IPM strategies are still commonly used by many conventional farmers [31], and given increased consumer awareness of the benefits of IPM practice, adoption rates are likely to rise.

It is for these reasons that we reject the organic-conventional dichotomy and emphasize that, in order to optimize environmental sustainability, individual tactics must be evaluated for their environmental impact in the context of an integrated approach, and that policy decisions must be based on empirical data and objective risk-benefit analysis, not arbitrary classifications.

## Materials and Methods

### Selection of insecticides for inclusion in experiments

In May 2008, the Pest Management Centre at AAFC provided us with a list of 14 potential insecticides for inclusion in our experiments. We reviewed each insecticide and eliminated those which had the same mode of action as any other insecticide registered for use against soybean aphid in Canada, and then contacted the suppliers to assess the economic feasibility of using these insecticides in field crops. Two novel synthetic and two organic insecticides were identified to be tested for management of soybean aphid, and the two registered insecticides were included in the experiment as conventional controls. Experimental application rates for novel insecticides were developed in consensus with supplier companies (Table 1). Table S1 provides a complete list of insecticides considered for inclusion in this experiment, and the rationale for products selected.

### Determination of direct contact toxicity to natural enemies

Adults and larvae of multicoloured Asian ladybeetle *Harmonia axyridis* and adults of insidious flower bug *Orius insidiosus* were treated with formulated insecticides at the equivalent of 0.5, 1 and 2× field rate using an airbrush spray tower. The untreated control consisted of 1 mL of distilled water. Groups of insects (8–10) were anesthetized using CO_2_ then placed in a 50 mm glass Petri plate lined with a piece of 47 mm qualitative filter paper, treated using the spray tower, and then placed in post-treatment containers. Each insecticide-concentration combination was repeated four times. The spray tower was rinsed with acetone, then distilled water, between each application.

#### *Orius insidiosus* assays

*Orius insidiosus* adults were obtained from commercial suppliers (BioBest Biological Systems Canada and MGS Horticultural Inc.). Repetitions of 10 adult *O. insidiosus* were treated, and then placed, post-treatment, in 10 cm plastic Petri plates lined with filter paper moistened with distilled water, and containing 1–2 washed baby spinach leaves, and an excess of frozen *Ephistia* eggs (BioBest Biological Systems Canada) for food. Mortality was recorded at 18, 24 and 48h post treatment.

#### *Harmonia axyridis* adult assays

*Harmonia axyridis* were obtained from aggregations on buildings in Guelph, Ontario, Canada, and were reared in laboratory cultures using procedures described by Xue et al [26]. Repetitions of 10 adult *H. axyridis* were treated, and then placed in 10 cm plastic Petri plates lined with filter paper moistened with distilled water, and containing several barley leaves infested with bird-cherry oat aphid (Aphid Banker System; Plant Products, Brampton, Ontario, Canada), and an excess of frozen *Ephistia* eggs (BioBest Biological Systems Canada) for food. Mortality was recorded every 24h for 168 h (7 d).

#### *Harmonia axyridis* larvae assays

Second and third instar *H. axyridis* were obtained from the laboratory culture described above. Assays were performed as adult assays above, except repetitions consisted of 8 individuals and instead of being placed together in a Petri plate, were placed individually into cells of a rearing tray (BIO-RT-32, C-D International, Inc.) with *Ephistia* eggs and aphid-infested barley to avoid cannibalism.

#### Statistical analysis of bioassay data

Mortality data was normalized using the Henderson-Tilton adjustment [32], and subjected to a mixed model ANOVA accounting for concentration (relative to field rate), treatment, and assessment time. Assessment time was treated as a repeated measure in the analysis.

### Determination of field efficacy and selectivity

In 2009, four soybean fields in southwestern Ontario with aphid populations approaching the action threshold of 250 aphids per plant were identified in collaboration with government extension personnel in July and August, 2009. After obtaining permission from landowners, sites were assessed once weekly until aphid populations exceeded 250 aphids per plant. Upon reaching this threshold, field experiments were initiated. In our initial screening trial in 2008, treatments were applied to a single site with a moderate density of aphids (∼120 aphids per plant), due to low aphid populations across our region during that year.

Field experiments employed a RCBD consisting of four blocks of 15 3.7×15.2m beds, with 3 untreated controls per block (one for each tractor pass required), our six insecticides and six other products or formulations not reported in this study. Insecticides were applied using a Teejet Duo nozzle configuration with spray tips #TT11002 at a height of 50cm above the canopy. Spray pressure at the nozzle was 276 kPa and the tractor travelled at a ground speed of 9.7km/h. Fluid delivery rate was maintained at 187 L/ha for all treatments. 2–3 soybean plants were destructively sampled from each bed at each assessment, and assessments were completed 1) immediately before treatment, 2) one week after treatment and 3) two weeks after treatment. Total numbers of aphids, ladybeetles, lacewings, parasitized aphid mummies, syrphid larvae, and flower bugs were assessed on each plant.

Aphid counts were transformed using Henderson-Tilton adjustments to account for population changes in the control between time of treatment and time of assessment, then subjected to a mixed model ANOVA accounting for site, year, tractor pass, replicate, and treatment.

### Field Selectivity Calculation

Field selectivity of each insecticide was estimated by calculating the change in the ratio of natural enemies to aphids in each plot, and subjecting these data to a mixed model ANOVA as above. We defined field selectivity as the relative change in the natural-enemy-to-pest population ratio observed after treatment. We standardized the counts of natural enemies of different species by defining a Natural Enemy Unit (NEU), where 1 NEU is the number of predators or parasitoids required to kill 100 pest insects in 24h. Thus,
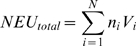
where N is the total number of natural enemy species, *n_i_* is the total number of individuals of natural enemy species *i* observed on 10 plants, and *V_i_* is the average voracity of natural enemy species *i*, that is, the number of pest insects it can kill in 24 h divided by 100. Using functional response data obtained by Xue et al. [26], we defined our soybean aphid ecosystem specific calculation as:
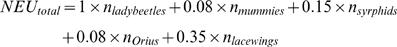
where *n_ladybeetles_* is the total number of adult and larvae of ladybeetles of *Harmonia axyridis* or *Coccinella septempunctata*, *n_mummies_* is the total number of parasitized aphids, *n_syrphids_* is the total number of Syrphidae larvae, *n_Orius_* is the total number of *Orius* spp., and *n_lacewings_* is the total number of Chrysopidae observed on 10 soybean plants.

Field selectivity was defined as the ratio of NEU/Aphids (NEU/A) after treatment to NEU/A before treatment, normalized by the control, as in the Henderson-Tilton adjustment [32], and took the form:
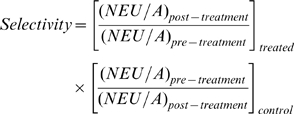



This selectivity index results in values <1 if a treatment kills more natural enemies than target pests, and values >1 if a treatment kills more target pests than natural enemies. Larger numbers will indicate a more target-selective pesticide. The selectivity index assumes the applied treatment has at least some efficacy against the target pest.

### Environmental Impact Assessment

EIQs were estimated using established methodology [29], [33] incorporating data from MSDS sheets provided by the supplier of the insecticides, an EIQ-field use rating was calculated for each insecticide, using the assumption that one application at field rate per season would provide equivalent aphid control. See Table 3 for values used in the calculation of EIQ for *Beauveria bassiana*, which does not have an existing published EIQ value.

**Table 3:** Toxicity ratings used to calculate Environmental Impact Quotient for *Beauveria bassiana*, which does not have a published EIQ value.

	Variables from EIQ Equation*										
	DT	D	F	Z	L	R	S	SY	C	P	B
**Active ingredient**	Dermal toxicity	Bird toxicity	Fish toxicity	Bee toxicity	Leaching potential	Runoff potential	Soil residue half life	Mode of action	Chronic health effects	Plant surface half life	Toxicity to beneficials
*Beauveria bassiana*	1	1	1	3	1	1	3	1	1	1	5

*Ratings were developed in accordance with methodology presented in Kovach et al. [29].

## Supporting Information

Table S1 Complete list of insecticides under consideration provided by Agriculture and Agri-Food Canada (AAFC).
(0.04 MB DOC)
