# Supplementary material for: Choosing Organic Pesticides over Synthetic Pesticides May Not Effectively Mitigate Environmental Risk in Soybeans
Source: PLoS One. 2010 Jun 22;5(6):e11250. doi: 10.1371/journal.pone.0011250 (PMC2889831; doi:10.1371/journal.pone.0011250)
Supplement: Table S1 — Complete list of insecticides under consideration provided by Agriculture and Agri-Food Canada (AAFC). (0.04 MB DOC) [file pone.0011250.s001.doc]

Table S1: Complete list of insecticides under consideration provided by Agriculture and Agri-Food Canada (AAFC)

| **Active ingredient (trade name, supplier)** | **Mechanism of action** | **Comments from AAFC*** | **Evaluation** | **Include in trial?** |
| --- | --- | --- | --- | --- |
| HGW86 (N/A, Dupont) | Unknown | Presented by the company at the Minor Use Meeting | No interest from supplier | No |
| Pymetrozine (Fulfil,® Syngenta) | Affects neuromuscular junctions | Reviewed as Reduced Risk (RR) pesticide for aphid control in US. Registered in Canada for aphids in potatoes but not soybeans | No interest from supplier | No |
| Spirotetramat (Movento®, Bayer) | Fatty acid biosynthesis inhibitor | Reviewed as RR product by IR-4/US. Potential for registration in legume vegetable crop group, soybean, etc | Novel MOA in soybeans, interest from supplier | Yes |
| *Beauveria bassiana* (Botanigard®, Laverlam) | Entomo-pathogenic fungus | Biopesticide | Novel MOA in soybeans, interest from supplier, can be used in organic-certified crops | Yes |
| Imidacloprid (Admire®, Bayer) | Acetylcholine agonist | Reviewed as OP replacement by IR-4/US | Same class of insecticide as seed treatments registered in soybean. | No |
| mineral oil (Superior 70 Oil®, UAP) | Oxygen exchange | - | Novel MOA in soybeans, interest from supplier, can be used in organic-certified crops | Yes |
| MOI-201plant extract (Marrone Organic Innovations) | Unknown | Botanical, see attached presentation | No interest from supplier | No |
| Flonicamid (Beleaf ®, ISK) | Neurotoxin- affects potassium channels | Reviewed as OP replacement by IR-4/US | Novel MOA in soybeans, interest from supplier | Yes |
| Clothianidin (Poncho®, Bayer) | Acetylcholine agonist | Reviewed as RR and OP replacement product by IR-4/US | Same class of insecticide as seed treatments registered in soybean. | No |
| Acetamiprid (Assail®, Nisso America Inc.) | Acetylcholine agonist | Reviewed as RR and OP replacement product by IR-4/US | Same class of insecticide as seed treatments registered in soybean. | No |
| Thiamethoxam (Actara®, Syngenta) | Acetylcholine agonist | Reviewed as OP replacement by IR-4/US | Same class of insecticide as seed treatments registered in soybean. | No |
| Cinnamaldehyde (Proguard®, Proguard Inc.) | Unknown | Natural product IR-4/US registered in soybean in US | Product has been discontinued | No |
| Spinetoram (Delegate®, Dow AgroSciences) | Acetylcholine agonist (but not at neonicotinoid site) | Reviewed as RR product by IR-4/US. | No interest from supplier | No |
| *Chrysoperla carnea* (Kagetaro®, Arysta Lifesciences) | Predator | Biopesticide IR-4/US (pepper & strawberries) | Not compatible with foliar spray technologies | No |

* OP = organophosphorus insecticide, RR= reduced risk pesticide, IR-4/US= “Interregional project # 4”- an ongoing pesticide risk reduction program commissioned by the United States Department of Agriculture.
